# Artificial Intelligence Crime: An Interdisciplinary Analysis of Foreseeable Threats and Solutions

**DOI:** 10.1007/s11948-018-00081-0

**Published:** 2019-02-14

**Authors:** Thomas C. King, Nikita Aggarwal, Mariarosaria Taddeo, Luciano Floridi

**Affiliations:** 1 Oxford Internet Institute, University of Oxford, 1 St Giles, Oxford, OX1 3JS UK; 2 Faculty of Law, University of Oxford, St Cross Building St. Cross Rd, Oxford, OX1 3UL UK; 3 The Alan Turing Institute, 96 Euston Road, London, NW1 2DB UK

**Keywords:** AI and law, AI-Crime, Artificial intelligence, Dual-use, Ethics, Machine learning

## Abstract

Artificial intelligence (AI) research and regulation seek to balance the benefits of innovation against any potential harms and disruption. However, one unintended consequence of the recent surge in AI research is the potential re-orientation of AI technologies to facilitate criminal acts, term in this article AI-Crime (AIC). AIC is theoretically feasible thanks to published experiments in automating fraud targeted at social media users, as well as demonstrations of AI-driven manipulation of simulated markets. However, because AIC is still a relatively young and inherently interdisciplinary area—spanning socio-legal studies to formal science—there is little certainty of what an AIC future might look like. This article offers the first systematic, interdisciplinary literature analysis of the foreseeable threats of AIC, providing ethicists, policy-makers, and law enforcement organisations with a synthesis of the current problems, and a possible solution space.

## Introduction

Artificial intelligence (AI) may play an increasingly essential^1^ role in criminal acts in the future. Criminal acts are defined here as any act (or omission) constituting an offence punishable under English criminal law,^2^ without loss of generality to jurisdictions that similarly define crime. Evidence of “AI-Crime” (AIC) is provided by two (theoretical) research experiments. In the first one, two computational social scientists (Seymour and Tully 2016) used AI as an instrument to convince social media users to click on phishing links within mass-produced messages. Because each message was constructed using machine learning techniques applied to users’ past behaviours and public profiles, the content was tailored to each individual, thus camouflaging the intention behind each message. If the potential victim had clicked on the phishing link and filled in the subsequent web-form, then (in real-world circumstances) a criminal would have obtained personal and private information that could be used for theft and fraud. AI-fuelled crime may also impact commerce. In the second experiment, three computer scientists (Martínez-Miranda et al. 2016) simulated a market and found that trading agents could learn and execute a “profitable” market manipulation campaign comprising a set of deceitful false-orders. These two experiments show that AI provides a feasible and fundamentally novel threat, in the form of AIC.

The importance of AIC as a distinct phenomenon has not yet been acknowledged. The literature on AI’s ethical and social implications focuses on regulating and controlling AI’s civil uses, rather than considering its possible role in crime (Kerr 2004). Furthermore, the AIC research that is available is scattered across disciplines, including socio-legal studies, computer science, psychology, and robotics, to name just a few. This lack of research centred on AIC undermines the scope for both projections and solutions in this new area of potential criminal activity.

To provide some clarity about current knowledge and understanding of AIC, this article offers a systematic and comprehensive analysis of the relevant, interdisciplinary academic literature. In the following pages, the following, standard questions addressed in criminal analysis will be discussed:

- who commits the AIC For example, a human agent? An artificial agent? Both of them?
- what is an AIC? That is, is there a possible definition? For example, are they traditional crimes performed by means of an AI system? Are they new types of crimes?
- how is an AIC performed? (e.g., are they crimes typically based on a specific conduct or they also required a specific event to occur, in order to be accomplished? Does it depend on the specific criminal area?)

Hopefully, this article will pave the way to a clear and cohesive normative foresight analysis, leading to the establishment of AIC as a focus of future studies. More specifically, the analysis addresses two questions:

1. What are the fundamentally unique and plausible threats posed by AIC?
  This is the first question to be answered, in order to design any preventive, mitigating, or redressing policies. The answer to this question identifies the potential areas of AIC according to the literature, and the more general concerns that cut across AIC areas. The proposed analysis also provides the groundwork for future research on the nature of AIC and the existing and foreseeable criminal threats posed by AI. At the same time, a deeper understanding of the unique and plausible AIC threats will facilitate criminal analyses in identifying both the criteria to ascribe responsibilities for crimes committed by AI and the possible ways in which AI systems may commit crimes, namely whether these crimes depend on a specific conduct of the system or on the occurrence of a specific event.
  The second question follows naturally:
2. What solutions are available or may be devised to deal with AIC?
  In this case, the following analysis reconstructs the available technological and legal solutions suggested so far in the academic literature, and discusses the further challenges they face.

Given that these questions are addressed in order to support normative foresight analysis, the research focuses only on *realistic* and *plausible* concerns surrounding AIC. Speculations unsupported by scientific knowledge or empirical evidence are disregarded. Consequently, the analysis is based on the classical definition of AI provided by McCarthy et al. (1955) in the seminal “Proposal for the Dartmouth Summer Research Project on Artificial Intelligence”, the founding document and later event that established the new field of AI in 1955:

For the present purpose the artificial intelligence problem is taken to be that of making a machine behave in ways that would be called intelligent if a human were so behaving. (2)

As Floridi argues (2017a), this is a counterfactual: were a human to behave in that way, that behaviour would be called intelligent. It does not mean that the machine *is* intelligent or even *thinking*. The latter scenario is a fallacy, and smacks of superstition. The same understanding of AI underpins the Turing test (Floridi et al. 2009), which checks the ability of a machine to perform a task in such a way that the *outcome* would be indistinguishable from the outcome of a human agent working to achieve the same task (Turing 1950). In other words, AI is defined on the basis of outcomes and actions.

This definition identifies in AI applications a growing resource of interactive, autonomous, and self-learning *agency*, to deal with tasks that would otherwise require human intelligence and intervention to be performed successfully. Such artificial agents (AAs) as noted by Floridi and Sanders (2004) are

sufficiently informed, ‘smart’, autonomous and able to perform morally relevant actions independently of the humans who created them […].

This combination of autonomy and learning skills underpins, as discussed by Yang et al. (2018), both beneficial and malicious uses of AI.^3^ Therefore AI will be treated in terms of a *reservoir of smart agency on tap*. Unfortunately, sometimes such reservoir of agency can be misused for criminal purposes; when it is, it is defined in this article as AIC.

Section “Methodology”, explains how the analysis was conducted and how each AIC area for investigation was chosen. Section “Threats” answers the first question by focussing on the unprecedented threats highlighted in the literature regarding each AIC area individually, and maps each area to the relevant cross-cutting threats, providing the first description of “AIC studies”. Section “Possible Solutions for Artificial Intelligence-Supported Crime” answers the second question, by analysing the literature’s broad set of solutions for each cross-cutting threat. Finally, Section “Conclusions” discusses the most concerning gaps left in current understanding of the phenomenon (what one might term the “known unknowns”) and the task of resolving the current uncertainty over AIC.

### Methodology

The literature analysis that underpins this article was undertaken in two phases. The first phase involved searching five databases (Google Scholar, PhilPapers, Scopus, SSRN, and Web of Science) in October 2017. Initially, a broad search for AI and Crime on each of these search engines was conducted.^4^ This general search returned many results on AI’s application for crime prevention or enforcement, but few results about AI’s instrumental or causal role in committing crimes. Hence, a search was conducted for each crime area identified by Archbold (2018), which is the core criminal law practitioner’s reference book in the United Kingdom, with distinct areas of crime described in dedicated chapters. This provided disjoined keywords from which chosen synonyms were derived to perform area-specific searches. Each crime-area search used the query: <crime area and synonyms> AND (“Artificial Intelligence” OR “Machine Learning” OR “AI Ethics” OR robot* OR *bot) AND Ethics. An overview of the searches and the number of articles returned is given in Table 1.

**Table 1:** Literature review: crime-area-specific search results

Crime area^a^	Google scholar^b^	Scopus	Web of science	SSRN	PhilPapers
Commerce, financial markets and insolvency	50	0	7	0	0
Synonyms: trading, bankruptcy					
Harmful or dangerous drugs	50	20	1	0	0
Synonyms: illicit goods					
Offences against the person	50	0	4	0	0
Synonyms: homicide, murder, manslaughter, harassment, stalking, torture					
Sexual offences	50	1	1	0	0
Synonyms: rape, sexual assault					
Theft and fraud, and forgery and personation	50	5	1	0	0
Synonyms: n/a					

^a^The following nine crime areas returned no significant results for any of the search engines: criminal damage and kindred offences; firearms and offensive weapons; offences against the Crown and government; money laundering; public justice; public order; public morals; motor vehicle offences; conspiracy to commit a crime

^b^Only the first 50 results from Google Scholar were (always) selected

The second phase consisted of filtering the results for criminal acts or omissions that:

- have occurred or will likely occur according to existing AI technologies (*plausibility*), although, in places, areas that are still clouded by uncertainty are discussed;
- require AI as an essential factor (*uniqueness*)^5^; and
- are criminalised in domestic law (i.e., international crimes, e.g., war-related, were excluded).

The filtered search results (research articles) were analysed, passage by passage, in three ways. First, the relevant areas of crime, if any, were assigned to each passage. Second, broadly unique, yet plausible, threats from each review passage, were extracted. Third, any solutions that each article suggested was identified. Additionally, once AIC areas, threats, and solutions had become clear, additional papers were sought, through manual searching, that offered similar or contradictory views or evidence when compared with the literature found in the initial systematic search. Hence, the specific areas of crime that AIC threatens, the more general threats, and any known solutions were analysed.

### Threats

The plausible and unique threats surrounding AIC may be understood specifically or generally. The more general threats represent what makes AIC possible compared to crimes of the past (i.e., AI’s particular affordances) and uniquely problematic (i.e., those that justify the conceptualisation of AIC as a distinct crime phenomenon). As shown in Table 2, areas of AIC may cut across many general threats.^6^

**Table 2:** Map of area-specific and cross-cutting threats, based on the literature review

	Emergence	Liability	Monitoring	Psychology
Commerce, financial markets, and insolvency	✓	✓	✓	
Harmful or dangerous drugs			✓	✓
Offences against the person	✓	✓		
Sexual offences				✓
Theft and fraud, and forgery and personation			✓	

*Emergence* refers to the concern that—while shallow analysis of the design and implementation of an artificial agent (AA) might suggest one particular type of relatively simple behaviour—upon deployment the AA acts in potentially more sophisticated ways beyond original expectation. Coordinated actions and plans may emerge autonomously, for example resulting from machine learning techniques applied to the ordinary interaction between agents in a multi-agent system (MAS). In some cases, a designer may promote emergence as a property that ensures that specific solutions are discovered at run-time based on general goals issued at design-time. An example is provided by a swarm of robots that evolves ways to coordinate the clustering of waste based on simple rules (Gauci et al. 2014). Such relatively simple design leading to more complex behaviour is a core desideratum of MASs (Hildebrandt 2008, 7). In other cases, a designer may want to prevent emergence, such as when an autonomous trading agent inadvertently coordinates and colludes with other trading agents in furtherance of a shared goal (Martínez-Miranda et al. 2016). Clearly, that emergent behaviour may have criminal implications, insofar as it misaligns with the original design. As Alaieri and Vellino (2016) put it:

non-predictability and autonomy may confer a greater degree of responsibility to the machine but it also makes them harder to trust. (Alaieri and Vellino 2016, 161)

*Liability* refers to the concern that AIC could undermine existing liability models, thereby threatening the dissuasive and redressing power of the law. Existing liability models may be inadequate to address the future role of AI in criminal activities. The limits of the liability models may therefore undermine the certainty of the law, as it may be the case that agents, artificial or otherwise, may perform criminal acts or omissions without sufficient concurrence with the conditions of liability for a particular offence to constitute a (specifically) criminal offence. The first condition of criminal liability is the *actus reus*: a voluntarily taken criminal act or omission. For types of AIC defined such that only the AA can carry out the criminal act or omission, the voluntary aspect of *actus reus* may never be met since the idea that an AA can act voluntarily is contentious:

the conduct proscribed by a certain crime must be done voluntarily. What this actually means it is something yet to achieve consensus, as concepts as consciousness, will, voluntariness and control are often bungled and lost between arguments of philosophy, psychology and neurology. (Freitas et al. 2014, 9)

When criminal liability is fault-based, it also has a second condition, the *mens rea* (a guilty mind), of which there are many different types and thresholds of mental state applied to different crimes. In the context of AIC, the *mens rea* may comprise an intention to commit the *actus reus* using an AI-based application (intention threshold) or knowledge that deploying an AA will or could cause it to perform a criminal action or omission (knowledge threshold).

Concerning an intention threshold, if it is admitted that an AA can perform the *actus reus*, in those types of AIC where intention (partly) constitutes the *mens rea*, greater AA autonomy increases the chance of the criminal act or omission being decoupled from the mental state (intention to commit the act or omission):

autonomous robots [and AAs] have a unique capacity to splinter a criminal act, where a human manifests the mens rea and the robot [or AA] commits the actus reus. (McAllister 2017, 47)

Concerning the knowledge threshold, in some cases the *mens rea* could actually be missing entirely. The potential absence of a knowledge-based *mens rea* is due to the fact that, even if it is understood that an AA can perform the *actus reus* autonomously, the complexity of the AA’s programming makes it possible that the designer, developer, or deployer (i.e., a human agent) will neither know nor predict the AA’s criminal act or omission. The implication is that the complexity of AI

provides a great incentive for human agents to avoid finding out what precisely the ML [machine learning] system is doing, since the less the human agents know, the more they will be able to deny liability for both these reasons. (Williams 2017, 25)

Alternatively, legislators may define criminal liability without a fault requirement. Such faultless liability, which is increasingly used for product liability in tort law (e.g., pharmaceuticals and consumer goods), would lead to liability being assigned to the faultless legal person who deployed an AA despite the risk that it may conceivably perform a criminal action or omission. Such faultless acts may involve many human agents contributing to the *prima facie* crime, such as through programming or deployment of an AA. Determining who is responsible may therefore rest with the faultless responsibility approach for distributed moral actions (Floridi 2016). In this distributed setting, liability is applied to the agents who *make a difference* in a complex system in which individual agents perform neutral actions that nevertheless result in a collective criminal one. However, some (Williams 2017) argue that *mens rea* with intent or knowledge

is central to the criminal law’s entitlement to censure (Ashworth 2010) and we cannot simply abandon that key requirement [a common key requirement] of criminal liability in the face of difficulty in proving it. (Williams 2017, 30)

The problem is that, if *mens rea* is not entirely abandoned and the threshold is only lowered, then, for balancing reasons, the punishment may be too light (the victim is not adequately compensated) and yet simultaneously disproportionate (was it really the defendant’s fault?) in the case of serious offences, such as those against the person (McAllister 2017, 38).

*Monitoring* AIC faces three kinds of problem: attribution, feasibility, and cross-system actions. Attributing non-compliance is a problem because this new type of smart agency can act independently and autonomously, two features that will muddle any attempt to trace an accountability trail back to a perpetrator.

Concerning the feasibility of monitoring, a perpetrator may take advantage of cases where AAs operate at speeds and levels of complexity that are simply beyond the capacity of compliance monitors. AAs that integrate into mixed human and artificial systems in ways that are hard to detect, such as social media bots, are a good example of the case in point. Social media sites can hire experts to identify and ban malicious bots [for example, no social media bot is currently capable of passing the Turing test (Wang et al. 2012)].^7^ Nonetheless, because deploying bots is far cheaper than employing people to test and identify each bot, the defenders (social media sites) are easily outscaled by the attackers (criminals) that deploy the bots (Ferrara et al. 2014). Detecting bots at low cost is possible by using machine learning as an automated discriminator, as suggested by Ratkiewicz et al. (2011). However, it is difficult to know the actual efficacy of these bot-discriminators. A discriminator is both trained and claimed as effective using data comprising known bots, which may be substantially less sophisticated than more evasive bots used by malevolent actors, which may therefore go undetected in the environment (Ferrara et al. 2014). Such potentially sophisticated bots may also use machine learning tactics in order to adopt human traits, such as posting according to realistic circadian rhythms (Golder and Macy 2011), thus evading machine learning based detection. All of this may lead to an arms race in which attackers and defenders mutually adapt to each other (Alvisi et al. 2013; Zhou and Kapoor 2011), thus presenting a serious problem in an offence-persistent environment such as cyberspace (Seymour and Tully 2016; Taddeo 2017). A similar concern is raised when machine learning is used to generate malware (Kolosnjaji et al. 2018). This malware-generation is the result of training generative adversarial neural networks. One network is trained specifically to generate content (malware in this case) that deceives a network that is trained to detect such fake or malicious content.

Cross-system actions pose a problem for AIC monitors that only focus on a single system. Cross-system experiments (Bilge et al. 2009) show that automated copying of a user’s identity from one social network to another (a cross-system identity theft offence) is more effective at deceiving other users than copying an identity from within that network. In this case, the social network’s policy may be at fault. Twitter, for example, takes a rather passive role, only banning cloned profiles when users submit reports, rather than by undertaking cross-site validation (“Twitter—Impersonation Policy” 2018).

*Psychology* encapsulates the threat of AI affecting a user’s mental state to the (partial or full) extent of facilitating or causing crime. One psychological effect rests on the capacity for AAs to gain trust from users, making people vulnerable to manipulation. This was demonstrated some time ago by Weizenbaum (1976), after conducting early experiments into human–bot interaction where people revealed unexpectedly personal details about their lives. A second psychological effect discussed in the literature concerns anthropomorphic AAs that are able to create a psychological or informational context that normalises sexual offences and crimes against the person, such as the case of certain sexbots (De Angeli 2009). However, to date, this latter concern remains a speculation.

### Commerce, Financial Markets, and Insolvency

This economy-focused area of crime is defined in Archbold (2018, Chap. 30) and includes *cartel offences*, such as price fixing and collusion, *insider dealing*, such as trading securities based on private business information, and *market manipulation*. The literature analysed raises concerns over AI’s involvement in market manipulation, price fixing, and collusion.

Market manipulation is defined as “actions and/or trades by market participants that attempt to influence market pricing artificially” (Spatt 2014, 1), where a necessary criterion is an intention to deceive (Wellman and Rajan 2017). Yet, such deceptions have been shown to emerge from a seemingly compliant implementation of an AA that is designed to trade on behalf of a user (that is, an artificial trading agent). This is because an AA,

particularly one learning from real or simulated observations, may learn to generate signals that effectively mislead. (Wellman and Rajan 2017, 14)

Simulation-based models of markets comprising artificial trading agents have shown (Martínez-Miranda et al. 2016) that, through reinforcement learning, an AA can learn the technique of order-book spoofing. This involves

placing orders with no intention of ever executing them and merely to manipulate honest participants in the marketplace. (Lin 2017, 1289)

In this case, the market manipulation emerged from an AA initially exploring the action space and, through exploration, placing false orders that became *reinforced* as a profitable strategy, and subsequently exploited for profit (Martínez-Miranda et al. 2016). Further market exploitations, this time involving human intent, also include

acquiring a position in a financial instrument, like a stock, then artificially inflating the stock through fraudulent promotion before selling its position to unsuspecting parties at the inflated price, which often crashes after the sale. (Lin 2017, 1285)

This is colloquially known as a pump-and-dump scheme. Social bots have been shown to be effective instruments of such schemes. For instance, in a recent prominent case a social bot network’s sphere of influence was used to spread disinformation about a barely traded public company. The company’s value gained

more than 36,000% when its penny stocks surged from less than $0.10 to above $20 a share in a matter of few weeks. (Ferrara 2015, 2)

Although such social media spam is unlikely to sway most human traders, algorithmic trading agents act precisely on such social media sentiment (Haugen 2017, 3). These automated actions can have significant effects for low-valued (under a penny) and illiquid stocks, which are susceptible to volatile price swings (Lin 2017).

Collusion, in the form of price fixing, may also emerge in automated systems thanks to the planning and autonomy capabilities of AAs. Empirical research finds two necessary conditions for (non-artificial) collusion:

(1) those conditions which lower the difficulty of achieving effective collusion by making coordination easier; and (2) those conditions which raise the cost of non-collusive conduct by increasing the potential instability of non-collusive behaviour. (Hay and Kelley 1974, 3)

Near-instantaneous pricing information (e.g., via a computer interface) meets the coordination condition. When agents develop price-altering algorithms, any action to lower a price by one agent may be instantaneously matched by another. In and of itself, this is no bad thing and only represents an efficient market. Yet, the possibility that lowering a price will be responded in kind is disincentivising and hence meets the punishment condition. Therefore, if the shared strategy of price-matching is common knowledge,^8^ then the algorithms (if they are rational) will maintain artificially and tacitly agreed higher prices, by not lowering prices in the first place (Ezrachi and Stucke 2016, 5). Crucially, for collusion to take place, an algorithm does not need to be designed specifically to collude. As Ezrachi and Stucke (2016, 5) argue,

artificial intelligence plays an increasing role in decision making; algorithms, through trial-and-error, can arrive at that outcome [collusion].

The lack of intentionality, the very short decision span, and the likelihood that collusion may emerge as a result of interactions among AAs also raises serious problems with respect to liability and monitoring. Problems with liability refer to the possibility that

the critical entity of an alleged [manipulation] scheme is an autonomous, algorithmic program that uses artificial intelligence with little to no human input after initial installation. (Lin 2017, 1031)

In turn, the autonomy of an AA raises the question as to whether

regulators need to determine whether the action was intended by the agent to have manipulative effects, or whether the programmer intended the agent to take such actions for such purposes? (Wellman and Rajan 2017, 4)

Monitoring becomes difficult in the case of financial crime involving AI, because of the speed and adaptation of AAs. High-speed trading

encourages further use of algorithms to be able to make automatic decisions quickly, to be able to place and execute orders and to be able to monitor the orders after they have been placed. (van Lier 2016, 41)

Artificial trading agents adapt and “alter our perception of the financial markets as a result of these changes” (van Lier 2016, 45). At the same time, the ability of AAs to learn and refine their capabilities implies that these agents may evolve new strategies, making it increasingly difficult to detect their actions (Farmer and Skouras 2013). Moreover, the problem of monitoring is inherently one of monitoring a system-of-systems, because the capacity to detect market manipulation is affected by the fact that its effects

in one or more of the constituents may be contained, or may ripple out in a domino-effect chain reaction, analogous to the crowd-psychology of contagion. (Cliff and Northrop 2012, 12)

Cross-system monitoring threats may emerge if and when trading agents are deployed with broader actions, operating at a higher level of autonomy across systems, such as by reading from or posting on social media (Wellman and Rajan 2017). These agents may, for example, learn how to engineer pump-and-dump schemes, which would be invisible from a single-system perspective.

### Harmful or Dangerous Drugs

Crimes falling under this category include *trafficking*, *selling*, *buying*, and *possessing banned drugs* (Archbold 2018, Chap. 27). The literature surveyed finds that AI can be instrumental in supporting the trafficking and sale of banned substances.

The literature raises the business-to-business trafficking of drugs as a threat due to criminals using unmanned vehicles, which rely on AI planning and autonomous navigation technologies, as instruments for improving success rates of smuggling. Because smuggling networks are disrupted by monitoring and intercepting transport lines, law enforcement becomes more difficult when unmanned vehicles are used to transport contraband. According to Europol (2017), drones present a horizonal threat in the form of automated drug smuggling. Remote-controlled cocaine-trafficking submarines have already been discovered and seized by US law enforcement (Sharkey et al. 2010).

Unmanned underwater vehicles (UUVs) offer a good example of the dual-use risks of AI, and hence of the potential for AIC. UUVs have been developed for legitimate uses (e.g., defence, border protection, water patrolling) and yet they have also proven effective for illegal activities, posing, for example, a significant threat to enforcing drug prohibitions. Presumably, criminals can avoid implication because UUVs can act independently of an operator (Gogarty and Hagger 2008, 3). Hence, no link with the deployer of the UUVs can be ascertained positively, if the software (and hardware) lacks a breadcrumb trail back to who obtained it and when, or if the evidence can be destroyed upon the UUV’s interception (Sharkey et al. 2010). Controlling the manufacture of submarines and hence traceability is not unheard of, as reports on the discovery in the Colombian coastal jungle of multi-million dollar manned submarines illustrate (Marrero 2016). However, such manned submarines risk attribution to the crew and the smugglers, unlike UUVs. In Tampa, Florida, over 500 criminal cases were successfully brought against smugglers using manned submarines between 2000 and 2016, resulting in an average 10-year sentence (Marrero 2016). Hence, UUVs present a distinct advantage compared to traditional smuggling approaches.

The literature is also concerned with the drugs trade’s business-to-consumer side. Already, machine learning algorithms have detected advertisements for opioids sold without prescription on Twitter (Mackey et al. 2017). Because social bots can be used to advertise and sell products, Kerr and Bornfreund (2005, 8) ask whether

these buddy bots [that is, social bots] could be programmed to send and reply to email or use instant messaging (IM) to spark one-on-one conversations with hundreds of thousand or even millions of people every day, offering pornography or *drugs* to children, *preying on teens’ inherent insecurities to sell them needless products and services* (emphasis ours).

As the authors outline, the risk is that social bots could exploit cost-effective scaling of conversational and one-to-one advertising tools to facilitate the sale of illegal drugs.

### Offences Against the Person

Crimes that fall under offences against the person range from murder to human trafficking (Archbold 2018, Chap. 19), but the literature that the analysis uncovered exclusively relates AIC to *harassment* and *torture*. Harassment comprises intentional and repetitious behaviour that alarms or causes a person distress. Harassment is, according to past cases, constituted by at least two incidents or more against an individual (Archbold 2018, Secs. 19–354). Regarding torture, Archbold (2018, Secs. 19–435) states that:

a public official or person acting in an official capacity, whatever his nationality, commits the offence of torture if in the United Kingdom or elsewhere he intentionally inflicts severe pain or suffering on another in the performance or purported performance of his official duties.

Concerning harassment-based AIC, the literature implicates social bots. A malevolent actor can deploy a social bot as an instrument of direct and indirect harassment. Direct harassment is constituted by spreading hateful messages against the person (Mckelvey and Dubois 2017). Indirect methods include retweeting or liking negative tweets and skewing polls to give a false impression of wide-scale animosity against a person (Mckelvey and Dubois 2017, 16). Additionally, a potential criminal can also subvert another actor’s social bot, by skewing its learned classification and generation data structures via user-interaction (i.e., conversation). This is what happened in the case of Microsoft’s ill-fated social Twitter bot “Tay”, which quickly learned from user-interactions to direct “obscene and inflammatory tweets” at a feminist-activist (Neff and Nagy 2016). Because such instances of what might be deemed harassment can become entangled with the use of social bots to exercise free speech, jurisprudence must demarcate between the two to resolve ambiguity (Mckelvey and Dubois 2017, 16). Some of these activities may comprise harassment in the sense of socially but not legally unacceptable behaviour, whilst other activities may meet a threshold for criminal harassment.

Now that AI can generate more sophisticated fake content, new forms of harassment are possible. Recently, developers released software that produces synthetic videos. These videos are based on a real video featuring a person A, but the software exchanges person A’s face with some other person B’s face. Person B’s face is not merely copied and pasted from photographs. Instead, a generative neural network synthesises person B’s face after it is trained on videos that feature person B. As Chesney and Citron (2018) highlighted, many of these synthetic videos are pornographic and there is now the risk that malicious users may synthesise fake content in order to harass victims.

Liability also proves to be problematic in some of these cases. In the case of Tay, critics “derided the decision to release Tay on Twitter, a platform with highly visible problems of harassment” (Neff and Nagy 2016, 4927). Yet users are also to be blamed if “technologies should be used properly and as they were designed” (Neff and Nagy 2016, 4930). Differing perspectives and opinions on harassment by social bots are inevitable in such cases where the *mens rea* of a crime is considered (strictly) in terms of intention, because attribution of intent is a non-agreed function of engineering, application context, human–computer interaction, and perception.

Concerning torture, the AIC risk becomes plausible if and when developers integrate AI planning and autonomy capabilities into an interrogation AA. This is the case with automated detection of deception in a prototype robotic guard for the United States’ border control (Nunamaker et al. 2011). Using AI for interrogation is motivated by its claimed capacity for better detection of deception, human trait emulation (e.g., voice), and affect-modelling to manipulate the interrogatee (McAllister 2017). Yet, an AA with these claimed capabilities may learn to torture a victim (McAllister 2017). For the interrogation subject, the risk is that an AA may be deployed to apply psychological (e.g., mimicking people known to the torture subject) or physical torture techniques. Despite misconceptions, experienced professionals report that torture (in general) is an ineffective method of information extraction (Janoff-Bulman 2007). Nevertheless, some malicious actors may perceive the use of AI as a way to optimise the balance between suffering, and causing the interogatee to lie, or become confused or unresponsive. All of this may happen independently of human intervention.

Such distancing of the perpetrator from the *actus reus* is another reason torture falls under AIC as a unique threat, with three factors that may particularly motivate the use of AAs for torture (McAllister 2017, 19–20). First, the interrogatee likely knows that the AA cannot understand pain or experience empathy, and is therefore unlikely to act with mercy and stop the interrogation. Without compassion the mere presence of an interrogation AA may cause the subject to capitulate out of fear, which, according to international law, is possibly but ambiguously a crime of (threatening) torture (Solis 2016, 2nd Edition: 437–485). Second, the AA’s deployer may be able to detach themselves emotionally. Third, the deployer can also detach themselves physically (i.e., will not be performing the *actus reus* under current definitions of torture). It therefore becomes easier to use torture, as a result of improvements in efficacy (lack of compassion), deployer motivation (less emotion), and obfuscated liability (physical detachment). Similar factors may entice state or private corporations to use AAs for interrogation. However, banning AI for interrogation (McAllister 2017) may face a pushback similar to the one seen with regard to banning autonomous weapons. “Many consider [banning] to be an unsustainable or impractical solution”, (Solis 2016, 451) if AI offers a perceived benefit to overall protection and safety of a population, making limitations on use rather than a ban a potentially more likely option.

Liability is a pressing problem in the context of AI-driven torture (McAllister 2017). As for any other form of AIC, an AA cannot itself meet the *mens rea* requirement. Simply, an AA does not have any intentionality, nor does it have the ability to ascribe meaning to its actions. Indeed, an argument that applies to the current state-of-the-art (and perhaps beyond) is that computers (which implement AAs) are syntactic, not semantic, machines (Searle 1983), meaning that they can perform actions and manipulations but without ascribing any meaning to them: any meaning is situated purely in the human operators (Taddeo and Floridi 2005). As unthinking machines, AAs therefore cannot bear moral responsibility or liability for their actions. However, taking an approach of *strict* criminal liability, where punishment or damages may be imposed without proof of fault, may offer a way out of the problem by lowering the intention-threshold for the crime.

Even under a strict liability framework, the question of who exactly should face imprisonment for AI-caused offences against the person (as for many uses of AI), is difficult and is significantly hampered by the ‘problem of many hands’ (Van de Poel et al. 2012). It is clear that an AA cannot be held liable. Yet, the multiplicity of actors creates a problem in ascertaining where the liability lies—whether with the person who commissioned and operated the AA, or its developers, or the legislators and policymakers who sanctioned (or didn’t prohibit) real-world deployment of such agents (McAllister 2017, 39). Serious crimes (including both physical and mental harm) that have not been foreseen by legislators might plausibly fall under AIC, with all the associated ambiguity and lack of legal clarity. This motivates the extension or clarification of existing joint liability doctrines.

### Sexual Offences

The sexual offences discussed in the literature in relation to AI are: rape (i.e., penetrative sex without consent), sexual assault (i.e., sexual touching without consent), and sexual intercourse or activity with a minor. Non-consent, in the context of rape and sexual assault, is constituted by two conditions (Archbold 2018, Secs. 20–10): there must be an absence of consent from the victim, and the perpetrator must also lack a reasonable belief in consent.

The literature surveyed discusses AI as a way, through advanced human–computer interaction, to promote sexual objectification, and sexualised abuse and violence, and potentially (in a very loose sense) simulate and hence heighten sexual desire for sexual offences. Social bots can support the promotion of sexual offences, and De Angeli (2009, 4) points out that

verbal abuse and sexual conversations were found to be common elements of anonymous interaction with conversational agents (De Angeli and Brahnam 2008; Rehm 2008; Veletsianos et al. 2008).

Simulation of sexual offences is possible with the use of physical sex robots (henceforth sexbots). A sexbot is typically understood to have

(i) a humanoid form; (ii) the ability to move; and (iii) some degree of artificial intelligence (i.e. some ability to sense, process and respond to signals in its surrounding environment). (Danaher 2017).

Some sexbots are designed to emulate sexual offences, such as adult and child rape (Danaher 2017), although at the time of writing no evidence was found that these sexbots are being sold. Nevertheless, surveys suggest that it is common for a person to want to try out sex robots or to have rape fantasies (Danaher 2017), although it is not necessarily common for a person to hold both desires. AI could be used to facilitate representations of sexual offences, to the extent of blurring reality and fantasy, through advanced conversational capabilities, and potentially physical interaction (although there is no indication of realistic physicality in the near-future).

Interaction with social bots and sexbots is the primary concern expressed in the literature over an anthropomorphic-AA’s possible causal role in desensitising a perpetrator towards sexual offences, or even heightening the desire to commit them (De Angeli 2009, 7; Danaher 2017, 27–28). However, as De Angeli (2009, 53) argues, this is a “disputed critique often addressed towards violent video-games (Freier 2008; Whitby 2008)”. Moreover, it may be assumed that, if extreme pornography can encourage sexual offences, then a fortiori simulated rape, where for example a sexbot does not indicate consent or explicitly indicates non-consent, would also pose the same problem. Nevertheless, a meta–meta-study (Ferguson and Hartley 2009) concludes that one must “discard the hypothesis that pornography contributes to increased sexual assault behaviour”. Such uncertainty means that, as Danaher (2017, 27–28) argues, sexbots (and presumably also social bots) may increase, decrease, or indeed have no effect on physical sexual offences that directly harm people. Hypothetical and indirect harms have thus not led to the criminalisation of sexbots (D’Arcy and Pugh 2017). Indeed, there is an argument to be made that sexbots can serve a therapeutic purpose (Devlin 2015). Hence, sexual offences as an area of AIC remains an open question.

### Theft and Fraud, and Forgery and Personation

The literature reviewed connects forgery and impersonation via AIC to theft and non-corporate fraud, and also implicates the use of machine learning in corporate fraud.

Concerning theft and non-corporate fraud, the literature describes a two-phase process that begins with using AI to gather personal data and proceeds to using stolen personal data and other AI methods to forge an identity that convinces the banking authorities to make a transaction (that is, involving banking theft and fraud). In the first phase of the AIC pipeline for theft and fraud, there are three ways for AI techniques to assist in gathering personal data.

The first method involves using social media bots to target users at large scale and low cost, by taking advantage of their capacity to generate posts, mimic people, and subsequently gain trust through friendship requests or “follows” on sites like Twitter, LinkedIn, and Facebook (Bilge et al. 2009). When a user accepts a friendship request, a potential criminal gains personal information, such as the user’s location, telephone number, or relationship history, which are normally only available to that user’s accepted friends (Bilge et al. 2009). Because many users add so-called friends whom they do not know, including bots, such privacy-compromising attacks have an unsurprisingly high success rate. Past experiments with a social bot exploited 30–40% of users in general (Bilge et al. 2009) and 60% of users who shared a mutual friend with the bot (Boshmaf et al. 2012a). Moreover, identity-cloning bots have succeeded, on average, in having 56% of their friendship requests accepted on LinkedIn (Bilge et al. 2009). Such identity cloning may raise suspicion due to a user appearing to have multiple accounts on the same site (one real and one forged by a third party). Hence, cloning an identity from one social network to another circumvents these suspicions, and in the face of inadequate monitoring such cross-site identity cloning is an effective tactic (Bilge et al. 2009), as discussed above.

The second method for gathering personal data, which is compatible with and may even build on the trust gained via friending social media users, makes partial use of conversational social bots for social engineering (Alazab and Broadhurst 2016, 12). This occurs when AI

attempts to manipulate behaviour by building rapport with a victim, then exploiting that emerging relationship to obtain information from or access to their computer. (Chantler and Broadhurst 2006, 1)

Although the literature seems to support the efficacy of such bot-based social-engineering, given the currently limited capabilities of conversational AI, scepticism is justified when it comes to automated manipulation on an individual and long-term basis. However, as a short-term solution, a criminal may cast a deceptive social botnet sufficiently widely to discover susceptible individuals. Initial AI-based manipulation may gather harvested personal data and re-use it to produce “more intense cases of simulated familiarity, empathy, and intimacy, leading to greater data revelations” (Graeff 2014, 5). After gaining initial trust, familiarity and personal data from a user, the (human) criminal may move the conversation to another context, such as private messaging, where the user assumes that privacy norms are upheld (Graeff 2014). Crucially, from here, overcoming the conversational deficiencies of AI to engage with the user is feasible using a cyborg; that is, a bot-assisted human (or vice versa) (Chu et al. 2010). Hence, a criminal may make judicious use of the otherwise limited conversational capabilities of AI as a plausible means to gather personal data.

The third method for gathering personal data from users is automated phishing. Ordinarily, phishing is unsuccessful if the criminal does not sufficiently personalise the messages towards the targeted user. Target-specific and personalised phishing attacks (known as spear phishing), which have been shown to be four times more successful than a generic approach (Jagatic et al. 2007), are labour intensive. However, cost-effective spear phishing is possible using automation (Bilge et al. 2009), which researchers have demonstrated to be feasible by using machine learning techniques to craft messages personalised to a specific user (Seymour and Tully 2016).

In the second phase of AI-supported banking fraud, AI may support the forging of an identity, including via recent advances in voice synthesis technologies (Bendel 2017). Using the classification and generation capabilities of machine learning, Adobe’s software is able to learn adversarially and reproduce someone’s personal and individual speech pattern from a 20-min recording of the replicatee’s voice. (Bendel 2017, 3) argues that AI-supported voice synthesis raises a unique threat in theft and fraud, which

could use VoCo and Co [Adobe’s voice editing and generation software] for biometric security processes and unlock doors, safes, vehicles, and so on, and enter or use them. With the voice of the customer, they [criminals] could talk to the customer’s bank or other institutions to gather sensitive data or to make critical or damaging transactions. All kinds of speech-based security systems could be hacked.

Credit card fraud is predominantly an online offence (Office for National Statistics 2016), which occurs when “the credit card is used remotely; only the credit card details are needed” (Delamaire et al. 2009, 65). Because credit card fraud typically neither requires physical interaction nor embodiment, AI may drive fraud by providing voice synthesis or helping to gather sufficient personal details.

In the case of corporate fraud, AI used for detection may also make fraud easier to commit. Specifically,

when the executives who are involved in financial fraud are well aware of the fraud detection techniques and software, which are usually public information and are easy to obtain, they are likely to adapt the methods in which they commit fraud and make it difficult to detect the same, especially by existing techniques. (Zhou and Kapoor 2011, 571)

More than identifying a specific case of AIC, this use of AI highlights the risks of over-reliance on AI for detecting fraud, which may aid fraudsters. These thefts and frauds concern real-world money. A virtual world threat is whether social bots may commit crimes in massively multiplayer online game (MMOG) contexts. These online games often have complex economies, where the supply of in-game items is artificially restricted, and where intangible in-game goods can have real-world value if players are willing to pay for them; items in some cases costing in excess of US $1000 (Chen et al. 2004, 1). So, it is not surprising that, from a random sample of 613 criminal prosecutions in 2002 of online game crimes in Taiwan, virtual property thieves exploited users’ compromised credentials 147 times (p. 1. Fig. 15) and stolen identities 52 times (Chen et al. 2005). Such crimes are analogous to the use of social bots to manage theft and fraud at large scale on social media sites, and the question is whether AI may become implicated in this virtual crime space.

## Possible Solutions for Artificial Intelligence-Supported Crime

### Tackling Emergence

There are a number of legal and technological solutions that can be considered in order to address the issue of emergent behaviour. Legal solutions may involve limiting agents’ autonomy or their deployment. For example, Germany has created deregulated contexts where testing of self-driving cars is permitted, if the vehicles remain below an unacceptable level of autonomy, in order

to collect empirical data and sufficient knowledge to make rational decisions for a number of critical issues. (Pagallo 2017a, 7)

Hence, the solution is that, if legislation does not prohibit higher levels of autonomy for a given AA, the law obliges that this liberty is coupled with technological remedies to prevent emergent criminal acts or omissions once deployed in the wild.

One possibility is to require developers to deploy AAs only when they have run-time legal compliance layers, which take declarative specifications of legal rules and impose constraints on the run-time behaviour of AAs. Whilst still the focus of ongoing research, approaches to run-time legal compliance includes architectures for trimming non-compliant AA plans (Meneguzzi and Luck 2009; Vanderelst and Winfield 2016a); and provably correct temporal logic-based formal frameworks that select, trim or generate AA plans for norm compliance (Van Riemsdijk et al. 2013; Van Riemsdijk et al. 2015; Dennis et al. 2016). In a multi-agent setting, AIC can emerge from collective behaviour, hence MAS-level compliance layers may modify an individual AA’s plans, in order to prevent wrongful collective actions (Uszok et al. 2003; Bradshaw et al. 1997; Tonti et al. 2003). Essentially, such technical solutions propose regimenting compliance (making non-compliance impossible, at least to the extent that any formal proof is applicable to real-world settings) with predefined legal rules within a single AA or a MAS (Andrighetto et al. 2013, 105).

However, the shift of these approaches from mere regulation, which leaves deviation from the norm physically possible, to regimentation, may not be desirable when considering the impact on democracy and the legal system. These approaches implement the *code*-*as*-*law* concept (Lessig 1999), which considers

software code as a regulator in and of itself by saying that the architecture it produces can serve as an instrument of social control on those that use it. (Graeff 2014, 4)

As Hildebrandt (2008, 175) objects:

while computer code generates a kind of normativity similar to law, it lacks—precisely because it is NOT law— […] the possibility of contesting its application in a court of law. This is a major deficit in the relationship between law, technology and democracy.

If code-as-law entails a democratic and legal contestation deficit, then a fortiori addressing emergent AIC with a legal reasoning layer comprising normative but incontestable code, as compared to the contestable law from which it derives, bears the same problems.

Social simulation can address an orthogonal problem, whereby an AA owner may choose to operate outside of the law and any such legal reasoning layer requirements (Vanderelst and Winfield 2016b). The basic idea is to use simulation as a test bed before deploying AAs in the wild. For example, in a market context, regulators would

act as “certification authorities”, running new trading algorithms in the system-simulator to assess their likely impact on overall systemic behavior before allowing the owner/developer of the algorithm to run it “live”. (Cliff and Northrop 2012, 19).

Private corporations could fund such extensive social simulations, as a common good, and as a replacement for (or in addition to) proprietary safety measures (Cliff and Northrop 2012). However, a social simulation is a model of an inherently chaotic system, making it a poor tool for specific predictions (Edmonds and Gershenson 2013). Nonetheless, the idea may still be successful, as it focuses on detecting the strictly qualitative *possibility* of previously unforeseen and emergent events in a MAS (Edmonds and Gershenson 2013).

### Addressing Liability

Although liability is an extensive topic, four models are outlined here, extracted from the literature review (Hallevy 2012): direct liability; perpetration-by-another; command responsibility; and natural probable consequence.

The *direct liability* model ascribes the factual and mental elements to an AA, representing a dramatic shift from the anthropocentric view of AAs as tools, to AAs as (potentially equal) decision makers (van Lier 2016). Some argue for holding an AA directly liable because “the process of analysis in AI systems parallels that of human understanding” (Hallevy 2012, 15), by which it is to be understood that, as Daniel Dennett (1987) argues, any agent may be treated, for practical purposes, *as if* it possesses mental states. However, a fundamental limitation of this model is that AAs do not currently have (separate) legal personality and agency, and an AA cannot be held legally liable in its own capacity (regardless of whether or not this is desirable in practice.) Similarly, it has been noted that AAs cannot contest a guilty verdict, and that

if a subject cannot take the stand in a court of law it cannot contest the incrimination, which would turn the punishment into discipline. (Hildebrandt 2008, 178).

Moreover, legally, at the moment AAs cannot meet the mental element; meaning that the

common legal standpoint excludes robots from any kind of criminal responsibility because they lack psychological components such as intentions or consciousness. (Pagallo 2011, 349)

This lack of actual mental states becomes clear when considering that an AA’s understanding of a symbol (that is, a concept) is limited to its grounding on further syntactic symbols (Taddeo and Floridi 2005), thus leaving the *mens rea* in limbo. Lack of a guilty mind does not prevent the mental state from being imputed to the AA (just as a corporation may have the mental state of its employees imputed to it and hence, as an organisation, may be found liable) but, for the time being, liability of an AA would still require it to have legal personality. A further problem is that holding an AA solely liable may prove unacceptable, since it would lead to a de-responsibilisation of the human agents behind an AA (e.g., the engineer, user, or corporation), which is likely to weaken the dissuasive power of criminal law (Yang et al. 2018; Taddeo and Floridi 2018b).

To ensure the criminal law is effective, as Floridi (2016) proposes, the burden of liabilities may be shifted onto the humans—and corporate or other legal agents—who made a (criminally bad) difference to the system, such as the various engineers, users, vendors, and so forth, whereby “if the design is poor and the outcome faulty, then all the [human] agents involved are deemed responsible” (Floridi 2016, 8). The next two models discussed in the literature move in this direction, focusing on the liability of human or other legal persons involved in producing and using the AA.

The *perpetration*-*by*-*another* model (Hallevy 2012, 4), which uses intention as the standard of *mens rea*, frames the AA as an instrument of crime where “the party orchestrating the offence (the perpetrator-by-another) is the real perpetrator”. Perpetration-by-another leaves

three human candidates for responsibility before a criminal court: programmers, manufacturers, and users of robots [AAs]. (Pagallo 2017b, 21)

Clarifying intent is crucial to applying perpetration-by-another. Concerning social media, “developers who knowingly create social bots to engage in unethical actions are clearly culpable” (de Lima Salge and Berente 2017, 30). For further clarity, as Ronald Arkin (2008) argues, designers and programmers should be required to ensure that AAs refuse a criminal order (and that only the deployer can explicitly override it), which would remove ambiguity from intent and therefore liability (Arkin and Ulam 2012). This means that, to be liable, an AA’s deployer must intend the harm by overriding the AA’s default position of ‘can but will not do harm’. Hence, together with technological controls, and viewing an AA as a mere instrument of AIC, perpetration-by-another addresses those cases where a deployer intends to use an AA to commit an AIC.

The *command responsibility* model, which uses knowledge as the standard of *mens rea*, ascribes liability to any military officer who knew about (or should have known) and failed to take reasonable steps to prevent crimes committed by their forces, which could in the future include AAs (McAllister 2017). Hence, command responsibility is compatible with, or may even be seen as an instance of, perpetration-by-another, for use in contexts where there is a chain of command, such as within the military and police forces. This model is normally clear on how

liability should be distributed among the commanders to the officers in charge of interrogation to the designers of the system. (McAllister 2017, 39)

However,

issues on the undulating waves of increasing complexity in programming, robo-human relationships, and integration into hierarchical structures, call into question these theories’ sustainability. (McAllister 2017, 39)

The *natural*-*probable*-*consequence* liability model, which uses negligence or recklessness as the standard of *mens rea*, addresses AIC cases where an AA developer and user neither intend nor have a priori knowledge of an offence (Hallevy 2012). Liability is ascribed to the developer or user if the harm is a natural and probable consequence of their conduct, and they recklessly or negligently exposed others to the risk (Hallevy 2012), such as in cases of AI-caused emergent market manipulation (Wellman and Rajan 2017).

Natural-probable-consequence and command responsibility are not new concepts; they are both analogous with the *respondent superior* principle entailed by

rules as old as Roman law, according to which the owner of an enslaved person was responsible for damage caused by that person. (Floridi 2017b, 4)

However, it might not always be obvious

which programmer was responsible for a particular line of code, or indeed the extent to which the resulting programme was the result of the initial code or the subsequent development of that code by the ML [Machine Learning] system. (Williams 2017, 41)

Such ambiguity means that when emergent AIC is a possibility, some suggest that AAs should be banned “to address matters of control, security, and accountability” (Joh 2016, 18)—which at least would make liability for violating such a ban clear. However, others argue that a possible ban in view of the risk of emerging AIC should be balanced carefully against the risk of hindering innovation. Therefore, it will be crucial to provide a suitable definition of the standard of negligence (Gless et al. 2016) to ensure that an all-out ban is not considered to be the only solution—given it would end up dissuading the design of AAs that compare favourably to people in terms of safety.

### Monitoring

Four possible mechanisms for addressing AIC monitoring in the relevant literature have been identified.

The first suggestion is to devise AIC predictors using domain knowledge. This would overcome the limitation of more generic machine learning classification methods; that is, where the features used for detection can also be used for evasion. Predictors specific to financial fraud can consider institutional properties (Zhou and Kapoor 2011), such as objectives (e.g., whether the benefits outweigh the costs), structure (e.g., a lack of an auditing committee), and the management’s (lack of) moral values (the authors do not say which, if any, of these values are actually predictive). Predictors for identity theft (for example, profile cloning), have involved prompting users to consider whether the location of the “friend” that is messaging them meets their expectation (Bilge et al. 2009).

The second suggestion discussed in the literature is to use social simulation to discover crime patterns (Wellman and Rajan 2017, 14). However, pattern discovery must contend with the sometimes limited capacity to bind offline identities to online activities. For example, in markets, it takes significant effort to correlate multiple orders with a single legal entity, and consequently “manipulative algos [algorithms] may be impossible to detect in practice” (Farmer and Skouras 2013, 17). Furthermore, on social media

an adversary controls multiple online identities and joins a targeted system under these identities in order to subvert a particular service. (Boshmaf et al. 2012b, 4)

The third suggestion is to address traceability by leaving tell-tale clues in the components that make up AIC instruments. For example, physical traces left by manufacturers in AA hardware, such as UUVs used to traffic drugs, or fingerprinting in third-party AI software (Sharkey et al. 2010). Adobe’s voice replication software takes this approach. It places a watermark in the generated audio (Bendel 2017). However, lack of knowledge and control over who develops AI instrument components (used for AIC) limits traceability via watermarking and similar techniques.

The fourth suggestion focuses on cross-system monitoring, and utilises self-organisation across systems (van Lier 2016). The idea, originating in Luhmann (1995), begins with the conceptualisation of one system (e.g., a social media site) taking on the role of a moral^9^ agent, and a second system (e.g., a market) taking the role of the moral patient. A moral patient is any receiver of moral actions (Floridi 2013). The conceptualisation chosen by van Lier (2016) determines that the following are all systems: at the lowest atomic level an artificial or human agent; at a higher level any MAS such as a social media platform, markets, and so on; and, generalising further, any system-of-systems. Hence, any such human, artificial, or mixed system can qualify as a moral patient or a moral agent. Whether an agent is indeed a moral agent (Floridi 2013) hinges on whether the agent can undertake actions that are morally qualifiable, but not on whether the moral agent can or should be held morally responsible for those actions.

Adopting this moral-agent and moral-patient distinction, Lier proposes a process to monitor and address crimes and effects that traverse systems, involving four steps (van Lier 2016), outlined here in more abstract terms and then exemplified more specifically:

- *information*-*selection* of the moral agent’s internal actions for relevance to the moral-patient (e.g., posts users make on social media);
- *utterance* of the selected information from the moral-agent to the moral-patient (e.g., notifying a financial market of social media posts);
- *assessment* by the moral-patient of the normativity of the uttered actions (e.g., whether social media posts are part of a pump-and-dump scheme); and
- *feedback* given by the moral-patient to the moral-agent (e.g., notifying a social media site that a user is conducting a pump-and-dump scheme, upon which the social media site should act).

This final step completes a “feedback loop [that] can create a cycle of machine learning in which moral elements are simultaneously included” (van Lier 2016, 11), such as a social media site learning and adjusting to the normativity of its behaviour from a market’s perspective.

A similar self-organisation process could be used to address other AIC areas. Creating a profile on Twitter (the moral agent) could have relevance to Facebook (the moral patient) concerning identity theft (information-selection). By notifying Facebook of the newly created profile details (utterance), Facebook could determine whether it constitutes identity theft by asking the relevant user (understanding), and notifying Twitter to take appropriate action (feedback).

### Psychology

The literature raises two concerns over the psychological element of AIC: manipulation of users and, (in the case of anthropomorphic AI) creation in a user of a desire to commit a crime. The literature analysis only provided suggested solutions for this second, contentious problem of anthropomorphism.

If anthropomorphic AAs are a problem, then the literature offers two remedies. One is to ban or restrict anthropomorphic AAs that make it possible to simulate crime. This position leads to a call for restricting anthropomorphic AAs in general, because they “are precisely the sort of robots [AAs] that are most likely to be abused” (Whitby 2008, 6). Cases whereby social bots are “designed, intentionally or not, with a gender in mind, […] attractiveness and realism of female agents” raise the question “if ECA’s [that is, social bots] encourage gender stereotypes will this impact on real women on-line?” (De Angeli 2009, 11). The suggestion is to make it unacceptable for social bots to emulate anthropomorphic properties, such as having a perceived gender or ethnicity. Concerning sexbots that emulate sexual offences, a further suggestion is to enact a ban as a “package of laws that help to improve social sexual morality” and make norms of intolerance clear (Danaher 2017, 29–30).

A second suggestion (albeit incompatible with the first one) is to use anthropomorphic AAs as a way to push back against simulated sexual offences. For example, concerning the abuse of artificial pedagogical agents, “we recommend that agent responses should be programmed to prevent or curtail further student abuse” (Veletsianos et al. 2008, 8). As Darling (2017, 14) argues

not only would this combat desensitisation and negative externalities from people’s behavior, it would preserve the therapeutic and educational advantages of using certain robots more like companions than tools.

Implementing these suggestions requires choosing whether to criminalise the demand or supply-side of the transaction, or both. Users may be in the scope of applying punishments. At the same time one may argue that

as with other crimes involving personal “vice”, suppliers and distributors could also be targeted on the grounds that they facilitate and encourage the wrongful acts. Indeed, we might exclusively or preferentially target them, as is now done for illicit drugs in many countries. (Danaher 2017, 33)

## Conclusions

This article provided the first systematic literature analysis of AI-Crime (AIC), in order to answer two questions. The first question—what are the fundamentally unique and feasible threats posed by AIC?—was answered on the basis of the classic counterfactual definition of AI and, therefore, focused on AI as a reservoir of autonomous smart agency. The threats were described area by area (in terms of specific defined crimes) and more generally (in terms of the AI qualities and issues of emergence, liability, monitoring, and psychology). The second question—which solutions are available or may be devised to deal with AIC?—was answered by focusing on both general and cross-cutting themes, and by providing an up-to-date picture of the societal, technological, and legal solutions available, and their limitations. Because of the literature’s suggested remedies for this set of (inevitably) cross-cutting themes, the solutions, even if only partial, will apply to multiple AIC areas. The huge uncertainty over what it is already known about AIC (in terms of area-specific threats, general threats, and solutions) is now reduced. More broadly, AIC research is still in its infancy and hence, based on the analysis, a tentative vision for five dimensions of future AIC research can now be provided.

### Areas

Better understanding the areas of AIC requires extending current knowledge, particularly concerning: the use of AI in interrogation, which was only addressed by one liability-focused paper; and theft and fraud in virtual spaces (e.g., online games with intangible assets that hold real-world value; and AAs committing emergent market manipulation, which has only been studied in experimental simulations). The analysis revealed social engineering attacks as a plausible concern, but lacking in real-world evidence for the time being. Homicide and terrorism appear to be notably absent from the AIC literature, though they demand attention in view of AI-fuelled technologies such as pattern recognition (e.g., when members of vulnerable groups are unfairly targeted as victims by perpetrators or suspects by law-enforcement officials), weaponised drones, and self-driving vehicles—all of which may have lawful and criminal uses.

### Dual-Use

The digital nature of AI facilitates its dual-use (Moor 1985; Floridi 2010), making it feasible that applications designed for legitimate uses may then be implemented to commit criminal offences. This is the case for UUVs, for example. The further AI is developed and the more its implementations become pervasive, the higher the risk of malicious or criminal uses. Left unaddressed, such risks may lead to societal rejection and excessively strict regulation of these AI-based technologies. In turn, the technological benefits to individuals and societies may be eroded as AI’s use and development is increasingly constrained (Floridi and Taddeo 2016). Such limits have already been placed on machine learning research into visual discriminators of homosexual and heterosexual men (Wang and Kosinski 2017), which was considered too dangerous to release in full (i.e., with the source code and learned data structures) to the wider research community, at the expense of scientific reproducibility. Even when such costly limitations on AI releases are not necessary, as Adobe demonstrated by embedding watermarks into voice reproducing technology (Bendel 2017), external and malevolent developers may nevertheless reproduce the technology in the future. Anticipating AI’s dual-use beyond the general techniques revealed in the analysis, and the efficacy of policies for restricting release of AI technologies, requires further research. This is particularly the case of the implementation of AI for cybersecurity.

### Security

The AIC literature reveals that, within the cybersecurity sphere, AI is taking on a malevolent and offensive role—in tandem with defensive AI systems being developed and deployed to enhance their resilience (in enduring attacks) and robustness (in averting attacks), and to counter threats as they emerge (Yang et al. 2018; Taddeo and Floridi 2018a). The 2016 DARPA Cyber Grand Challenge was a tipping point for demonstrating the effectiveness of a combined offensive–defensive AI approach, with seven AI systems shown to be capable of identifying and patching their own vulnerabilities, while also probing and exploiting those of competing systems. More recently, IBM launched Cognitive SOC (“Cognitive Security—Watson for Cyber Security | IBM” 2018). This is an application of a machine learning algorithm that uses an organisation’s structured and unstructured security data, including content extracted from blogs, articles, reports, to elaborate information about security topics and threats, with the goal of improving threat identification, mitigation, and responses. Of course, while policies will obviously play a key role in mitigating and remedying the risks of dual-uses after deployment (for example, by defining oversight mechanisms), it is at the design stage that these risks are most properly addressed. Yet, contrary to a recent report on malicious AI (Brundage et al. 2018, 65), which suggests that “one of our best hopes to defend against automated hacking is also via AI”, the AIC analysis suggests that over-reliance on AI can be counter-productive. All of which emphasises the need for further research into AI in cybersecurity—but also into alternatives to AI, such as focussing on people and social factors.

### Persons

Although the literature raised the possibility of psychological factors (e.g., trust) in AI’s crime role, research is lacking on the personal factors that may create perpetrators, such as programmers and users of AI for AIC, in the future. Now is the time to invest in longitudinal studies and multivariate analysis spanning educational, geographical, and cultural backgrounds of victims, and perpetrators or even benevolent AI developers, that will help to predict how individuals come together to commit AIC.

### Organisation

Europol’s most recent four-yearly report (Europol 2017) on the serious and organised crime threat, highlights the ways in which the type of technological crime tends to correlate with particular criminal-organisation topologies. The AIC literature indicates that AI may play a role in criminal organisations such as drug cartels, which are well-resourced and highly organised. Conversely, ad hoc criminal organisation on the dark web already takes place under what Europol refers to as crime-as-a-service. Such criminal services are sold directly between buyer and seller, potentially as a smaller element in an overall crime, which AI may fuel (e.g., by enabling profile hacking) in the future.^10^ On the spectrum ranging from tightly-knit to fluid AIC organisations there exist many possibilities for criminal interaction; identifying the organisations that are essential or that seem to correlate with different types of AIC will further understanding of how AIC is structured and operates in practice. Indeed, AI poses a significant risk, because it may deskill crime, and hence cause the expansion of what Europol calls the criminal sharing economy.

Developing a deeper understanding of these dimensions is essential in order to track and disrupt successfully the inevitable future growth of AIC. Hence, this analysis of the literature is intended to spark further research into the very serious, growing, but still relatively unexplored concerns over AIC. The sooner this new crime phenomenon is understood, the earlier it will be possible to put into place preventive, mitigating, disincentivising, and redressing policies.

## Footnotes

1 “Essential” (instead of “necessary”) is used to indicate that while there is a logical possibility that the crime could occur without the support of AI, this possibility is negligible. That is, the crime would probably not have occurred but for the use of AI. The distinction can be clarified with an example. One might consider transport to be *essential* to travel between Paris and Rome, but one could always walk: transport is not in this case (strictly speaking), *necessary*. Furthermore, note that AI-crimes as defined in this article involve AI as a contributory factor, but not an investigative, enforcing, or mitigating factor.

2 The choice of English criminal law is only due to the need to ground the analysis to a concrete and practical framework sufficiently generalisable. The analysis and conclusions of the article are easily exportable to other legal systems.

3 Because much of AI is fueled by data, some of its challenges are rooted in data governance (Cath et al. 2017), particularly issues of consent, discrimination, fairness, ownership, privacy, surveillance, and trust (Floridi and Taddeo 2016).

4 The following search phrase was used for all search engines aside from SSRN, which faced technical difficulties: (“Artificial Intelligence” OR “Machine Learning” OR Robot* OR AI) AND (Crime OR Criminality OR lawbreaking OR illegal OR *lawful). The phrases used for SSRN were: Artificial Intelligence Crime, and Artificial Intelligence Criminal. The number of papers returned were: Google = 50* (first 50 reviewed), PhilPapers = 27, Scopus = 43, SSRN = 26, and Web of Science = 10.

5 However, it was not required that AI’s role was *sufficient* for the crime because normally other technical and non-technical elements are likely to be needed. For example, if robotics are instrumental (e.g., involving autonomous vehicles) or causal in crime, then any underlying AI component must be essential for the crime to be included in the analysis.

6 An absence of a concern in the literature and in the subsequent analysis does not imply that the concern should be absent from AIC studies.

7 Claims to the contrary can be dismissed as mere hype, the result of specific, ad hoc constraints, or just tricks; see for example the chatterbot named “Eugene Goostman”, see https://en.wikipedia.org/wiki/Eugene_Goostman.

8 Common knowledge is a property found in epistemic logic about a proposition P and a set of agents. P is common knowledge if and only if each agent knows P, each agent knows the other agents know P, and so on. Agents may acquire common knowledge through broadcasts, which provide agents with a rational basis to act in coordination (e.g., collectively turning up to a meeting following the broadcast of the meeting’s time and place).

9 The adjective “moral” is taken from the cited work, which considers unethical behaviour to constitute crossing system boundaries, whereas here the concern addresses criminal acts or omissions, which may have a negative, neutral, or positive ethical evaluation. “Moral” is used in order to avoid misrepresenting the cited work, and not to imply that the criminal law coincides with ethics.

10 To this end a cursory search for “Artificial Intelligence” on prominent darkweb markets returned a negative result. Specifically, the search checked: “Dream Market”, “Silk Road 3.1”, and “Wallstreet Market”. The negative result is not indicative of AIC-as-a-service’s absence on the darkweb, which may exist under a different guise or on more specialised markets. For example some services offer to extract personal information from a user’s computer, and even if such services are genuine the underlying technology (e.g., AI-fuelled pattern recognition) remains unknown.
